# Study on the Microscopic Mechanism of Enhanced Oil Recovery by Nano–Surfactant Flooding System in Low Permeability Reservoirs

**DOI:** 10.3390/ma19163383

**Published:** 2026-08-08

**Authors:** Peiwen Xiao, Kai Lv, Xiang Peng, Jie Li, Qun Zhang, Yuanping Lin, Yanqi Li, Weidong Liu, Yinzhu Ye

**Affiliations:** 1 Research Institute of Petroleum Exploration and Development, PetroChina, Beijing 100083, China; 2 State Key Laboratory of Enhanced Oil Recovery (Key Laboratory of Nano Chemistry), Beijing 100083, China; 3 Institute of Porous Flow and Fluid Mechanics, Chinese Academy of Sciences, Langfang 065007, China; 4 Yumen Oilfield Company, PetroChina, Jiuquan 735000, China; 5 Oil Production Technology Research Institute of Xinjiang Oilfield Company, PetroChina, Karamay 834000, China; 6 Oil Production Technology Research Institute of Yumen Oilfield Company, PetroChina, Jiuquan 735000, China

**Keywords:** low permeability reservoirs, nanofluid–surfactant binary system, enhanced oil recovery, nanofluid-flooding technology

## Abstract

This study addresses the limitations of traditional chemical flooding in low-permeability reservoirs by developing a nanofluid–surfactant binary flooding system (Has5/iNanoW1.0 binary flooding system) and investigating its microscopic mechanisms and enhanced oil recovery performance. The size of the nanofluid–surfactant binary flooding system reduces from 250 nm to 72 nm compared with pure surfactant; therefore it can enhance the ability to inject smaller pores. Core adsorption tests reveal that the addition of nanofluid can decrease surfactant adsorption by over 30%. The nanoscale synergistic effect of nanofluid (iNanoW1.0) and surfactant (Has5) and lower adsorption of surfactant allows more surfactant (Has5) to enter smaller pores for oil washing, significantly increasing oil recovery performance. Low-field nuclear magnetic resonance displacement experiments show that the binary system can significantly expand microscopic sweep efficiency (up to 14.9%) compared to pure surfactant or nanofluid flooding. Core flooding tests confirm that the binary system exhibits lower injection pressure (0.377 MPa), achieving 13.12% incremental oil recovery during post-water flooding, which is significantly better than the pure surfactant system (5.11%). The results demonstrate strong laboratory-scale potential and permit further pilot-scale evaluation.

## 1. Introduction

Recent years, the focus of world oil and gas development has gradually shifted to the exploitation of low-permeability reservoirs. However, such reservoirs generally have the characteristics of small pores and complex pore structure, which leads to the significant restriction of the performance of traditional chemical flooding technology [1]. With the rapid development of nanotechnology, nanomaterials have been widely applied in the oil industry, especially in the field of enhanced oil recovery [2,3,4,5]. The small size characteristic of nanomaterials increases the specific surface area of the material exponentially with the decrease of particle size, which significantly increases not only the number of surface-active sites, but also the chemical activity of surface functional groups [6]. These excellent physical and chemical properties provide important advantages for the application of nanomaterials in enhanced oil recovery.

The mechanism of nanofluid for enhance oil recovery has been widely reported, mainly including key mechanisms such as wettability regulation, interfacial tension reduction and structural separation pressure. For the wettability regulation mechanism, nanofluid can change the wettability of rock through surface adsorption. Specifically, nanoparticles can be adsorbed on the surface of the rock to change from lipophilic to hydrophilic, or bind to crude oil through the hydrophobic end to enhance the fluidity of crude oil [7,8,9]. Karimi et al. [10] studied the wettability changes of carbonate rocks and zirconia (ZrO_2_) nanofluids. Combined with microscopic imaging and theoretical analysis, it was found that due to the difference in oil–water two-phase partition coefficients, ZrO_2_ nanoparticles were preferentially deposited on the rock surface, thereby inducing wettability reversal. For interfacial tension reduction mechanism, nanoparticles can reduce oil–water interfacial tension to improve oil recovery. Wu et al. [11] used the Pickering emulsion method to hydrophobically modify SiO_2_ nanoparticles. The experiment showed that when the concentration of Janus nanoparticles reached 0.05 wt%, the oil–water interfacial tension could be reduced to 2.28 mN/m, which significantly improved the oil displacement efficiency. For structural separation pressure mechanism, in the three-phase (nanofluid–solid–liquid) contact region, nanoparticles can form an ordered wedge structure, and its local concentration is higher than the bulk concentration of the suspension. The resulting osmotic pressure promotes interface separation and forms an additional driving force, that is, structural separation pressure [12,13]. The study of Chang et al. [14] further revealed that the enrichment of hydrophilic nanoparticles in the three-phase contact region will enhance the structural separation pressure, while hydrophobic nanoparticles expand the contact region by increasing the interface thickness. The mixed nanoparticles are mainly distributed at the oil–water interface, which reduces the interfacial tension and promotes the detachment of oil droplets. Moreover, the dynamic behavior of nanoparticles in pore throat also has been studied as an important influence on the oil displacement process. Due to the density difference between nanoparticles and water, its deposition at the pore throat can lead to a local pressure rise, prompting the water flow to turn and drive the crude oil in the adjacent pore throat. After the crude oil is expelled, the flow resistance is reduced [15]. In the exploitation of heavy oil, nanoparticles can effectively inhibit asphaltene precipitation, and its mechanism of action is mainly two-fold: adsorption and dispersion [16]. Nanomaterials can absorb asphaltene molecules by virtue of high specific surface area and surface activity, and hinder asphaltene aggregation through intermolecular forces and steric hindrance effects, thereby improving the fluidity of heavy oil.

The Key Laboratory of Nanochemistry of PetroChina developed the nanofluid oil displacement agent iNanoW1.0 [17,18], which can effectively reduce the hydrogen bond association among water molecules and change the network structure of water, so that it can enter the low permeability area that cannot be entered by conventional water flooding, thereby expanding the swept volume. The ability to weaken the hydrogen bond association increases with the increase of the mass fraction of iNanoW1.0, and tends to be stable after reaching 0.10%. The research results lay a theoretical foundation for the use of a nano-oil displacement agent to expand the swept volume and provide an important theoretical and experimental reference for water injection development of low permeability-tight oil reservoirs. However, its oil washing ability needs to be further improved.

Surfactants play an irreplaceable role in oilfield development due to their excellent oil washing ability [18,19]. However, in practical applications, due to the complexity of reservoir conditions, surfactants are prone to a large amount of adsorption on the surface of formation rocks, which not only increases the cost of chemical agents, but also reduces their efficiency. The combination of nanomaterials and surfactants have been studied recently. Almahfood et al. [20] found that there is a strong interaction between nanoparticles and surfactant molecules, which can form a stable surfactant–nanoparticle composite system, to prepare a uniformly dispersed nanosuspension. This composite system can not only further reduce the oil–water interfacial tension, but also improve the wettability of nanoparticles through the modification of surfactants, and significantly improve the stability of nanofluids [21,22,23]. In practical applications, this composite system has shown significant advantages. Suleimanov et al. [24] combined nano-SiO_2_ dispersion with anionic surfactant for oil displacement in low permeability reservoirs, which increased the recovery rate by more than 30%. Cheng et al. [25] prepared AMPS/AM modified nano-SiO_2_ binary composite oil displacement agent by in-situ polymerization technology and verified the effectiveness of its enhanced oil recovery. The study of Li et al. [26] further revealed that nano-SiO_2_ can not only adsorb surfactant molecules to form a stable composite system but also produce a significant flow control effect by combining the hydrophilic properties of the surface functional groups with the particle aggregation effect. These studies showed that the combination of nanomaterials and surfactant can make nanomaterials more stable and that they have some possibility to be used as a flooding agent in enhanced oil recovery. However, these systems did not show any synergetic effects between nanomaterials and surfactants. There are still some key deficiencies in the research on the synergistic enhancement of oil recovery by nanomaterials and surfactants. Firstly, the interaction mechanism between the two has not been fully elucidated, especially the microscopic interaction between nanoparticles and surfactant molecules and its dynamic behavior mechanism at the oil–water–rock interface. Secondly, the existing experiments are mostly limited to macroscopic evaluation, and there is a lack of research on the long-term stability and migration law under real reservoir conditions. Furthermore, the large-scale preparation process, cost control and environmental safety assessment of the composite system still need to be further explored. Finally, the adaptability rules and engineering application specifications for different reservoir conditions have not yet been established.

This paper aims to solve the problems of difficult injection of ordinary water into low permeability reservoirs, low recovery rate of chemical flooding and major damage to the formation. The nanofluid–surfactant binary flooding system (Has5/iNanoW1.0 binary flooding system) developed in this paper can effectively improve recovery rate and reduce formation damage. The change of particle size was analyzed and discussed by laser particle size analyzer. The interfacial properties were investigated by interfacial tension test and contact angle test. Through the adsorption experiment, the change in the adsorption amount of the chemical agent in the formation with the addition of the nano oil displacement agent was discussed. Finally, enhanced oil recovery ability and fluid distribution were explored by low field nuclear magnetic displacement equipment.

## 2. Experimental

This section introduces the preparation of nanofluids, the steps in parameter characterization, the interface ability test, the design and method of adsorption experiment and the low field nuclear magnetic displacement experimental equipment.

### 2.1. Materials and Equipment

Heavy alkylbenzene sulfonate (Has5) (Lanzhou Refinery, 50% active ingredient, Lanzhou, China), iNanoW1.0 nanofluid (Xi’an Changqing Chemical Group Co., LTD, Xi’an, China) and deionized water (laboratory homemade).

In this paper, the surfactant is heavy alkylbenzene sulfonate (Has5), with nanofluid as the displacement agent (iNanoW1.0). The surfactant concentration used in this paper was fixed as 0.2 wt%, and the iNanoW1.0 concentrations were 0.01 wt%, 0.03 wt%, 0.06 wt%, 0.1 wt%, 0.2 wt% and 0.3 wt%. The nanofluid–surfactant binary flooding systems (Has5/iNanoW1.0) were prepared by diluting Has5 with deionized water and adding iNanoW1.0 with different concentrations separately, and the system was stirred at 500 r/min for 10 min.

### 2.2. Compatibility Experiments of Nanofluid-Surfactant Binary Flooding System

The compatibility study adopts macroscopic compatibility and microscopic compatibility. Macroscopic compatibility refers to placing the prepared nanofluid–surfactant binary flooding system in the oven at the temperature of the target reservoir and observing the precipitation of the respective precipitates. Microscopic compatibility refers to the injection of nanofluid–surfactant binary flooding system into a 0.5 mm capillary tube, sealed and insulated, and placed under a microscope to observe the precipitation of the nanofluid–surfactant binary flooding system in the capillary tube. At the same time, the particle size of the sample was measured by the particle size technique of the Zeta potential particle size tester.

### 2.3. Interfacial Tension Experiments

The interfacial tension of different nanofluid–surfactant binary flooding systems were measured by the spin-drop interfacial tension tester TX500C. The capillary tube was rinsed with the solution to be tested and the solution to be tested was filled with the capillary tube. Then the oil droplets were injected into the capillary tube and placed in the measuring instrument. The rotational speed and temperature were set to measure the interfacial tension for one hour.

### 2.4. Adsorption Experiments

In this experiment, the static adsorption method was used to test the adsorption amount between the chemical agent and the formation. In order to facilitate the measurement, the liquid–solid mass ratio is 10:1, that is, the surfactant solution is 10 g, and the surfactant concentration is 250 ppm to 5000 ppm (because the high concentration surfactant solution will not have good compatibility with the iNanoW1.0, the concentration is adjusted to 250 ppm to 3500 ppm after adding the iNanoW1.0). The conductivity value of surfactant solution with different concentrations was measured by conductivity meter and the standard curve of conductivity value of surfactant solution was drawn. Sand (1 g) with a particle size of 400 μm was added to the solution and shaken in a shaker for 24 h. Then the sand was separated from the surfactant solution using a centrifuge, and the supernatant was taken to measure its conductivity. The concentration was calculated according to the standard curve of the surfactant solution, and the difference between the initial concentration and the final concentration of the surfactant was determined. The adsorption capacity was calculated by Formula (1).

(1) $$A=\frac{\left[{(C}_{i}-C_{f})\times \frac{M_{s}}{M_{r}}\right]}{1000}$$

In the formula, A is the adsorption amount of surfactant, the unit is mg/g, $C_{i}$ and $C_{f}$ refer to the initial concentration and final concentration of surfactant solution, respectively, the unit is ppm, $M_{s}$ and $M_{r}$ refer to the mass of solution and the mass of sand added, respectively, and the unit is g.

Five groups of different surfactant solutions were prepared using 0 wt%, 0.2 wt%, 0.3 wt%, 0.4 wt% and 0.5 wt% iNanoW1.0 as raw materials. The difference in surfactant concentration was found by measuring the conductivity of the solution before and after sand removal. Formula (1) is used to calculate the adsorption amount of surfactant in the presence of iNanoW1.0. Different concentrations of surfactant solution were prepared. By measuring the conductivity before adsorption and after adsorption for 24 h, 48 h, 72 h and 96 h, the change in adsorption amount over time was calculated by Formula (1) through the difference in surfactant concentration.

### 2.5. Core Flooding Test with Nuclear Magnetic Resonance Experiment

The low field nuclear magnetic resonance core displacement device is mainly formed by the displacement device, nuclear magnetic resonance device, metering device and so on. The nuclear magnetic resonance (NMR) relaxation signal (*T*_2_ spectrum) of ^1^H proton-containing fluid in rock pores can be obtained by NMR measurement of rocks containing water (oil) using a low-field NMR core displacement device. Due to the distribution of pores of different sizes in the rock, the measured data are the result of the superposition of multiple transverse relaxation components. The obtained *T*_2_ spectrum reflects the spatial distribution of the ^1^H proton-containing fluid in the core. The larger the transverse relaxation time, the larger the pore diameter of the ^1^H proton-containing fluid. On the contrary, the smaller the transverse relaxation time, the smaller the pore diameter of the ^1^H proton-containing fluid. The peak area enclosed by the amplitude and relaxation time of the signal represents the total signal value of the ^1^H proton fluid in the core pore. The larger the peak area, the more ^1^H proton fluid in the core pore; the smaller the peak area, the less ^1^H proton fluid in the pore. The on-line detection principle of nuclear magnetic resonance is to use low-field nuclear magnetic technology to detect a series of *T*_2_ spectra in real time during the displacement process of ^1^H proton-containing fluid in the core. Based on the *T*_2_ spectrum, the real-time saturation of ^1^H proton-containing fluid in the core can be analyzed. Because the deuterium (^2^H) proton in deuterium water has no signal in low field nuclear magnetic resonance, deuterium water is used as the medium of saturated core, which can be distinguished from water. The experiment was carried out on the MesoMR23-060H-HTHP core nuclear magnetic analyzer (Shanghai Newmai Electronic Technology Co., Ltd., Shanghai, China). The main test parameters were: magnetic field strength 0.5 T, resonance frequency 21 MHz, probe coil diameter 70 mm, echo time 0.3 ms, recovery time 3000 ms, cumulative number 16, echo number 8000 and *T*_2_ spectrum fitting point 100.

### 2.6. Conventional Oil Displacement Experiments

**Figure 1:**
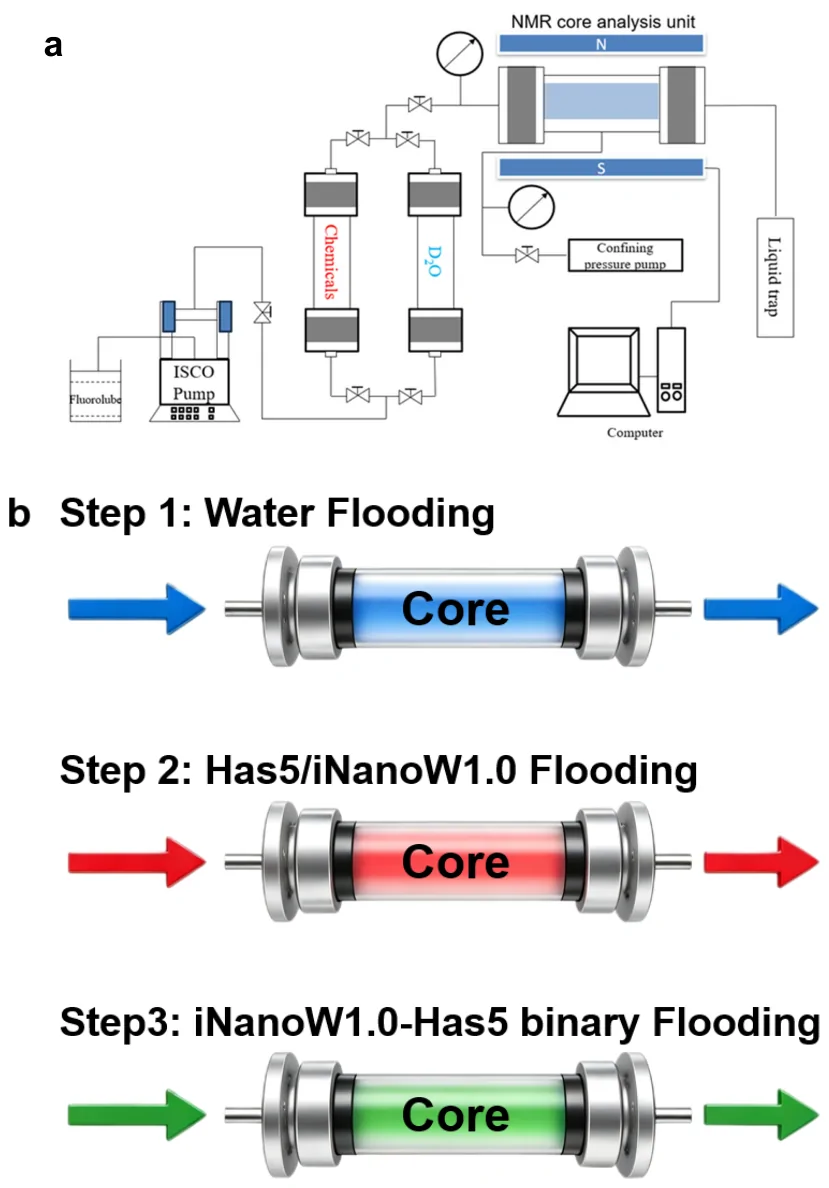
(**a**) Schematic diagram of low field nuclear magnetic resonance core displacement device and (**b**) core flooding test flowchart.

The conventional displacement experimental device (Figure 1a) was used to carry out the microemulsion enhanced oil recovery experiment and was compared with the performance of iNanoW1.0-Has5 nanofluid–surfactant binary flooding system. The pressure at different times and the volume changes of the oil phase and water phase in the produced fluid were recorded, and the displacement recovery was calculated. Experimental scheme (Figure 1b): 1. The core data used for displacement are shown in Table 1 and Table 2, the core processing and expansion are similar and the simulated oil is saturated; 2. Put the treated core into the core holder, empty the air in the holder with a double-cylinder pump and transport the ring pressure to 5 MPa. The double-cylinder pump is used to discharge the air at the inlet end of the gripper, close the emptying valve, set the displacement speed, open the outlet valve for water flooding and record the pressure and the volume change of the oil phase and the water phase in the liquid at intervals. When the water content reaches more than 98%, the water flooding is stopped. After water flooding is completed, it is connected to the inlet end of the gripper and emptied. After closing the emptying valve, the chemical agent displacement speed is set and the outlet valve is opened for chemical agent displacement and relevant data are recorded. After the chemical agent is injected into about 0.8 PV, it is converted into subsequent water flooding, and the relevant data are recorded. When the water content reaches more than 98%, the experiment is stopped; 3. The data are processed to obtain chemical and enhanced recovery, pressure changes and changes in water content.

## 3. Result and Discussion

This section introduces the change in iNanoW1.0 concentration on the morphology of the nanofluid–surfactant binary flooding system and the change in particle size. The effect of iNanoW1.0 on interfacial tension was studied. Secondly, the adsorption of surfactants and the influence of iNanoW1.0 on adsorption are introduced. At the same time, the change of time rule on the adsorption of surfactants is discussed. Finally, the effects of different iNanoW1.0 concentrations on extended sweep and enhanced oil recovery and their fluid distribution were studied by low-field nuclear magnetic resonance displacement technology.

### 3.1. Compatibility and Particle Size Results

**Figure 2:**
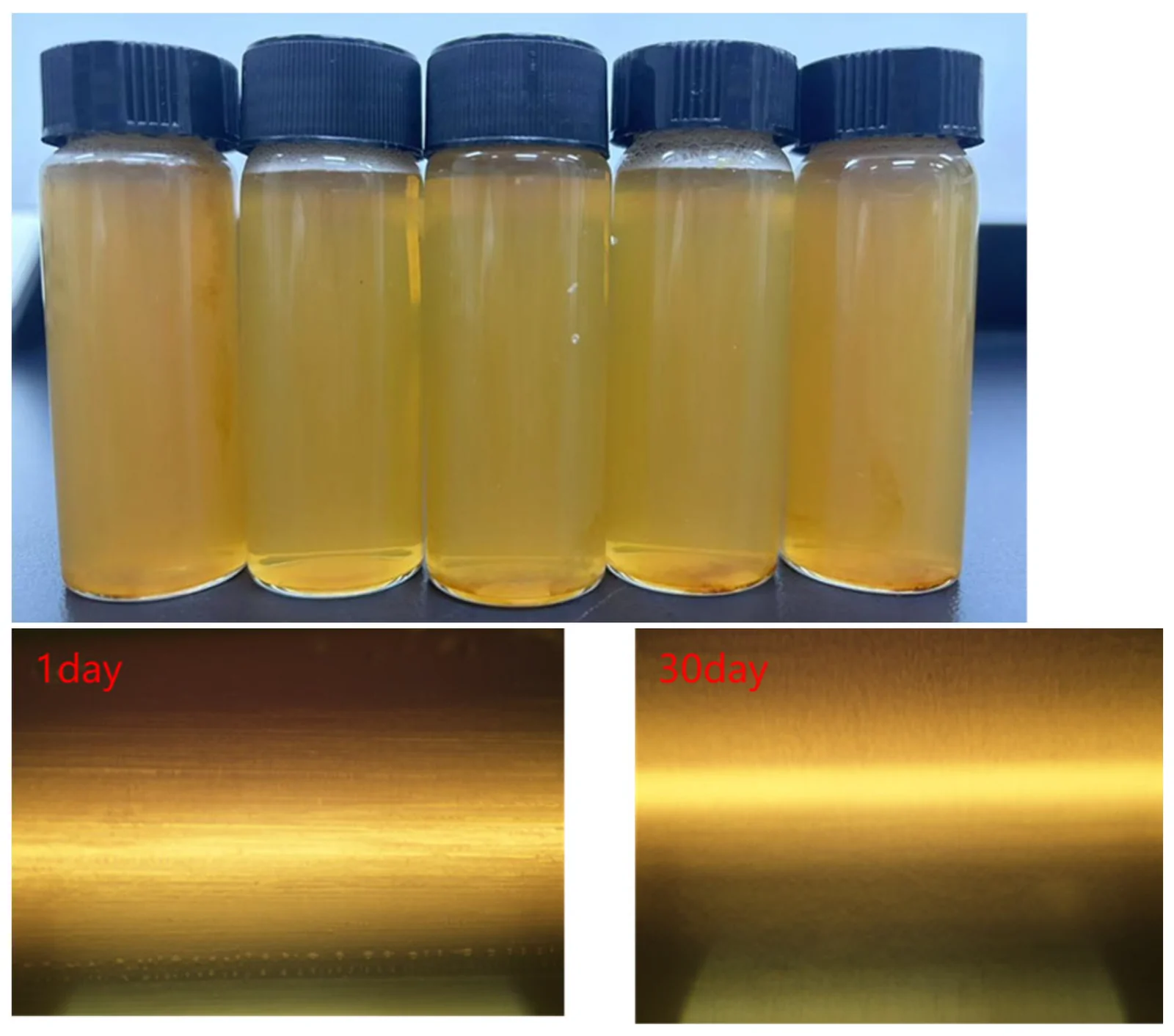
Taking 0.1% iNanoW-0.2% Has5 as an example, no precipitation occurred within 30 days.

As shown in Figure 2, after observing the compatibility of the nanofluid–surfactant binary flooding system, it was found that with the addition of iNanoW1.0, the compatibility of the system did not change significantly and there was no precipitation in the macro and micro systems. This proves that the heavy alkylbenzene sulfonate surfactant has good compatibility with iNanoW1.0 and is more suitable for synergistic use.

**Figure 3:**
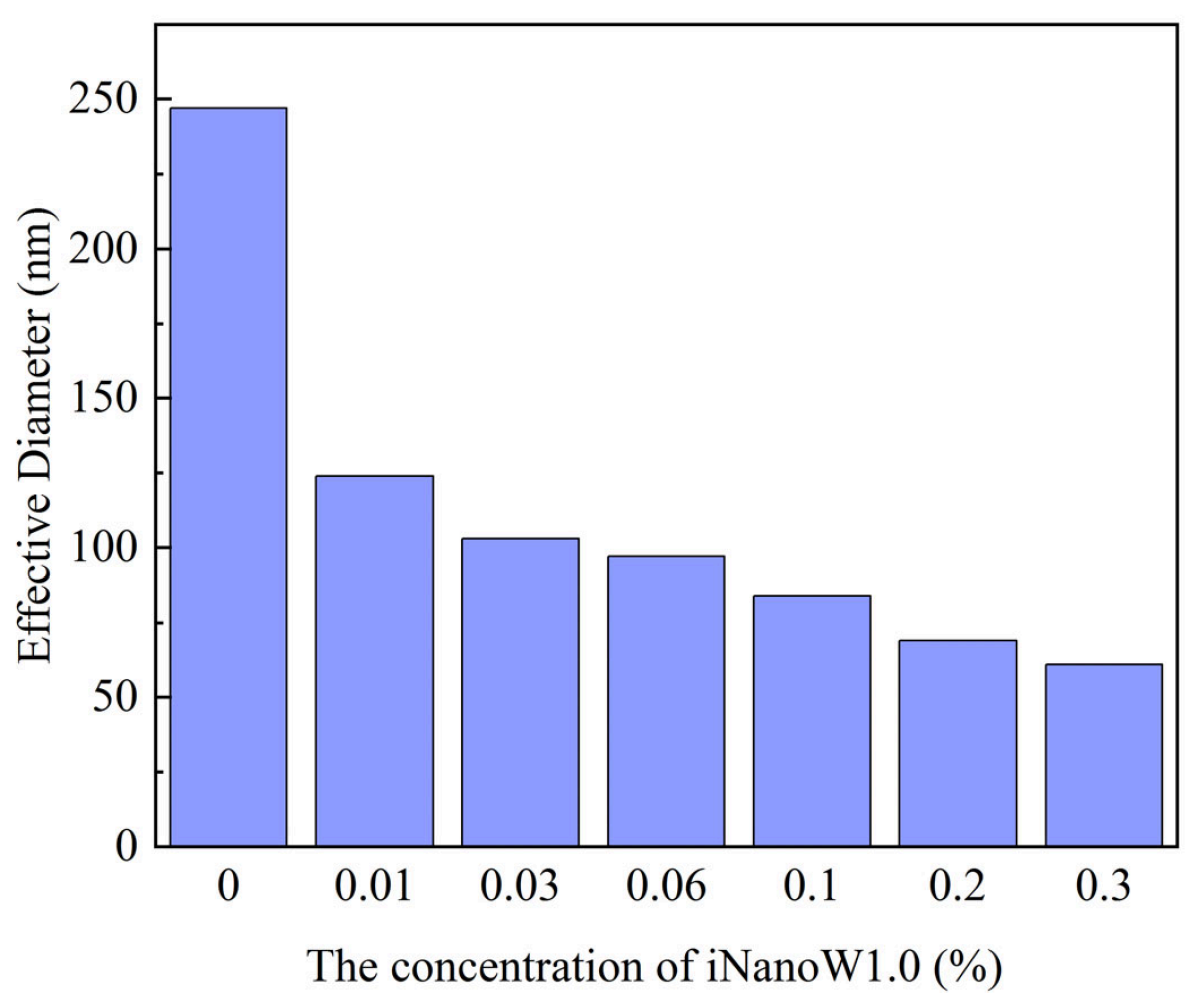
Variation of nanofluid–surfactant binary flooding system particle size.

As shown in Figure 3, with the increase of iNanoW1.0 concentration, the size of the system showed a downward trend.

The particle size of the system reached about 250 nm before the nano-agent was added, and there were intermolecular forces such as hydrogen bonds between the surfactant molecules. The surfactant itself assembles into a large micelle structure. With the addition of the nano-agent, the hydrogen bonds between the molecules are destroyed, and the larger micron-sized micelle structure is reduced to the nanometer level. And its size distribution is a single peak. When the concentration of nano-agent reaches 0.3%, the particle size of the system reaches a minimum of 72 nm, which can enter smaller pores to exert the oil displacement effect.

### 3.2. Interfacial Tension Test Results

**Figure 4:**
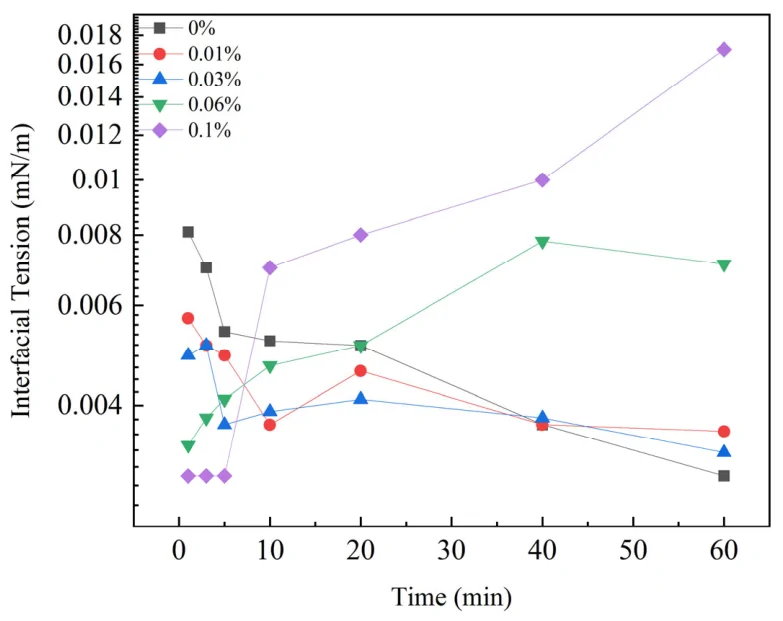
Change of interfacial tension.

Interfacial tension is a main factor affecting oil recovery for surfactant. The smaller the interfacial tension is, the easier it is for the displacement fluid to drive crude oil out of the pores, thereby improving oil recovery. Figure 4 shows the change of interfacial tension when the concentration of iNanoW1.0 changes, where the concentration of Has5 is fixed at 0.2 wt%.

The interfacial tension of Has5 surfactant with a single 0.2% concentration is 3 × 10^−3^ mN/m, but with the addition of iNanoW1.0, the interfacial tension of the system gradually increases. When the concentration of nano-agent is 0.1%, the interfacial tension of the system increases to 1.7 × 10^−2^ mN/m, which is higher than the ultra-low interfacial tension. Therefore, in the use of the synergistic system, attention should be paid to the influence of iNanoW1.0 on the interfacial tension of the system to maintain its ability to reduce the interfacial tension. It can be seen from the figure that when the concentration of the fixed surfactant is constant, unlike other types of surfactants, the oil–water interfacial tension of the system gradually increases with the increase of the concentration of iNanoW1.0 and does not decrease. Because the surfactant is adsorbed at the oil–water interface to achieve the effect of reducing the oil–water interfacial tension, and iNanoW1.0 may preferentially occupy the interface position, forming a physical barrier, and the surfactant and iNanoW1.0 are negatively charged, electrostatic repulsion will hinder the co-adsorption of the two. iNanoW1.0 is mainly modified by silica, and silica may enhance the mechanical strength of the interfacial film, but if the film structure is too rigid or disordered, it will lead to an increase in interfacial tension. A small amount of iNanoW1.0 can enhance the interfacial stability through synergistic effects. However, when the concentration of iNanoW1.0 is too high, they will accumulate excessively at the interface, causing steric hindrance and hindering the effective adsorption of surfactants. This is also one of the main reasons for the increase of interfacial tension with the increase of iNanoW1.0 concentration. When the concentration of iNanoW1.0 is fixed, the interfacial tension of the system gradually decreases with the increase of surfactant concentration, and it changes little when the surfactant concentration is 0.2% and 0.3%. Both can reduce the interfacial tension to ultra-low in a short time.

### 3.3. Adsorption Changes

**Figure 5:**
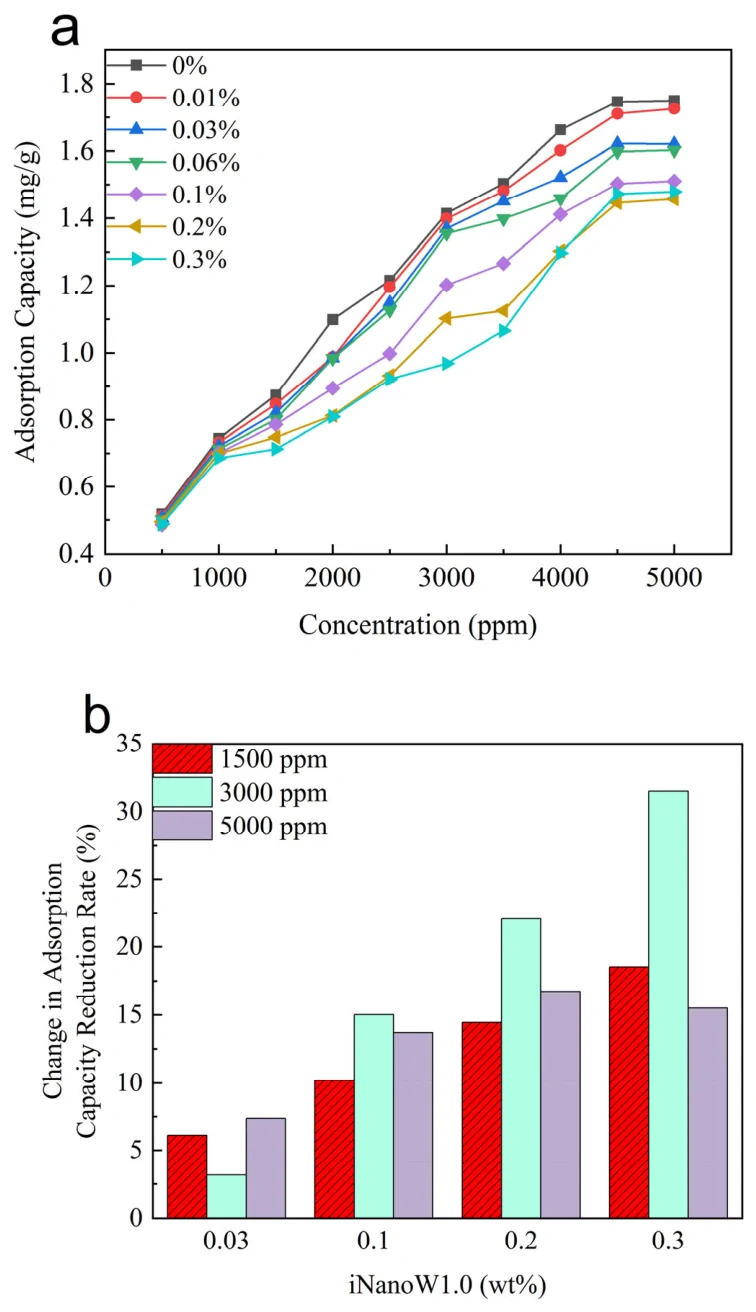
(**a**) Change of adsorption capacity, (**b**) change in adsorption capacity reduction rate.

In chemical flooding, adsorption is crucial. Reducing the adsorption of chemical agents in the formation will make chemical flooding more economically feasible and reduce the damage caused to the formation by chemical agents. As the concentration of iNanoW1.0 increases, the adsorption will decrease (Figure 5a). iNanoW1.0 of 0.01—0.3% concentration was added to the surfactant solution to understand its effect on the heavy alkylbenzene sulfonate surfactant on the surface of the adsorbed sand particles. When the concentration of Has5 is 500 ppm, the adsorption capacity of the surfactant is 0.5179 mg/g. As the concentration of Has5 increases, the adsorption capacity of the surfactant also increases gradually. When the concentration of Has5 is 5000 ppm, the adsorption capacity increases to 1.75 mg/g. The adsorption capacity decreased from 0.52 mg/g to 0.49 mg/g with the change of the concentration of iNanoW1.0 in the surfactant aqueous solution. When the concentration of Has5 is 5000 ppm, the adsorption capacity of the surfactant reaches the maximum value of 1.75 mg/g, but when the concentration of Has5 surfactant is 4500 ppm, the change in its adsorption capacity is not obvious. Because Has5 is an anionic surfactant, its molecular structure contains negatively charged hydrophilic groups; while the sand surface is weakly negatively charged, below 5000 ppm concentration, Has5 is more free molecules and more easily adsorbed. With the increase of surfactant concentration, the adsorption capacity also increases. When the surfactant concentration exceeds 4500 ppm, the adsorption of its free molecules in the formation is basically saturated, and it is not easy to adsorb in the formation. The adsorption is only affected by the free molecules of surfactants in the aqueous phase.

**Figure 6:**
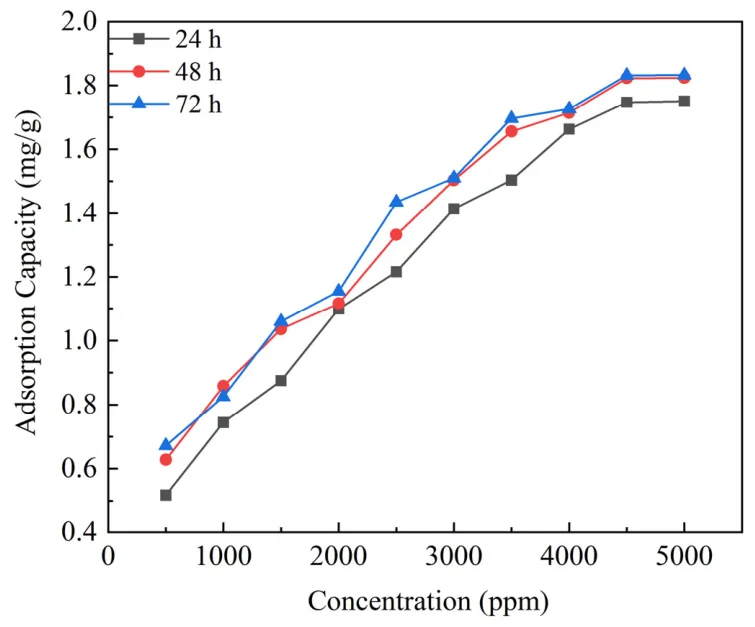
The change of Has5 adsorption capacity with time.

Adding iNanoW1.0 to the surfactant solution, the free molecules of the surfactant bind to the iNanoW1.0, thereby reducing the number of free molecules of the surfactant, thereby reducing the adsorption of Has5 in the sand. It can be seen from (Figure 5a) that even if the concentration of iNanoW1.0 continues to increase, the effect of reducing the adsorption amount will eventually stabilize, which indicates that the number of surfactant molecules bound to iNanoW1.0 is limited, similar to the adsorption of a single surfactant solution. When the concentration exceeds 4500 ppm, the adsorption amount will not continue to increase but will remain unchanged. Similarly, in the Has5 adsorption reduction, the adsorption reduction of the solution below 3000 ppm continues to increase. When the concentration of iNanoW1.0 increases from 0.01% to 0.3% (Figure 5b), and the surfactant concentration is 3000 ppm, the adsorption reduction increases from 27% to 46%. At the same iNanoW1.0 concentration, although the surfactant concentration increases, the adsorption reduction remains unchanged or even decreases. The presence of iNanoW1.0 can reduce surfactant adsorption for many reasons. Due to the weak negative charge of iNanoW1.0, the electrostatic repulsion between iNanoW1.0 and quartz sand is weak. Therefore, some iNanoW1.0 will be adsorbed on the surface of quartz sand and compete with surfactants to reduce the adsorption of surfactants. Moreover, the adsorption capacity of surfactants increases over time and also varies with their concentration (Figure 6). Within the same time interval, the incremental adsorption of surfactants gradually decreases, and eventually reaching adsorption equilibrium. As the adsorption time increases, the adsorption capacity of the chemical agent in the for-mation reaches saturation, so even if the adsorption time increases from 48 h to 72 h, the adsorption capacity doesn’t not change much.

### 3.4. Low Field Nuclear Magnetic Expanded Microscopic Volume Ripple Experiment

In the various stages of the core expansion micro-volume sweep experiment, the nuclear magnetic resonance technology was used to explore the fluid change law inside the core. In this experiment, two expanded microscopic volume sweep experiments were carried out. The first was water flooding, followed by 0.2% Has5 surfactant solution flooding and finally 0.2% Has5/0.03% iNanoW1.0. In the second and first comparison, the surfactant displacement in the second stage was changed to 0.03% iNanoW1.0nanofluid.

The above displacement experiments were carried out at a constant displacement rate of 0.1 mL/min. Firstly, CPMG data sampling was performed on the core after the deionized water was used to drive the water until the nuclear magnetic signal did not change significantly. CPMG data sampling was performed after 0.2% Has5 solution (or 0.03% iNanoW1.0) prepared by deionized water was displaced until the nuclear magnetic signal did not change significantly. Finally, the 0.2% Has5-0.3% iNanoW1.0 complex system prepared with deionized water was used to displace the NMR signal to no significant change and CPMG data sampling was performed.

**Figure 7:**
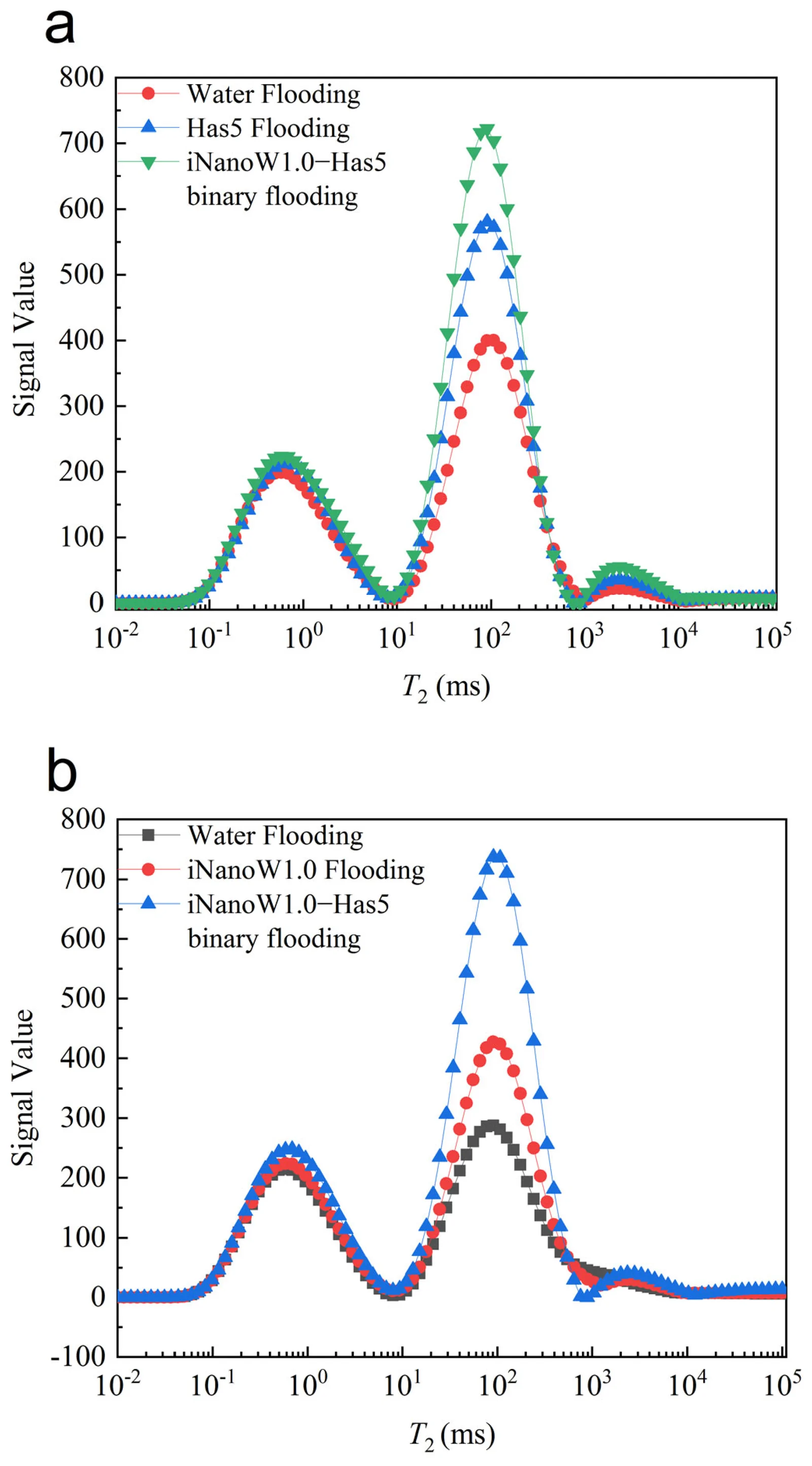
(**a**) Core 1 Expanded sweep experiment, (**b**) Core 2 Expanded sweep experiment.

The experimental results show that after water flooding, regardless of the use of surfactant flooding or nano-agent flooding, it will effectively expand the micro-volume sweep capacity. Among them, the use of surfactant flooding after water flooding increases the sweep volume by 13.4%; after water flooding, nano-agent flooding was used to further increase the swept volume by 9.2% (Figure 7a). The use of surfactant/nano-agent composite system displacement after surfactant or nano-agent displacement can also effectively expand its swept volume. The use of a composite system after surfactant displacement can increase its swept volume by 9.3%. After nano-agent flooding, the compound system was used to increase the swept volume by 14.9% (Figure 7b). Obviously, the composite system has more enhanced sweep efficiency after nano-agent displacement. This is because the particle size of the nanofluid is generally between 20–30 nm, and it does not play a major role in the mesopores. The particle size of the composite system is about 100 nm, which can more effectively expand the sweep volume of the mesopores.

### 3.5. Core Displacement Experiment

In the field of enhanced oil recovery, short core displacement experiment is an important means to verify whether the chemical agent is effective or not. In this part, two groups of core displacement experiments were carried out. The first group was water flooding; after the water content reached 98%, the second stage was replaced by surfactant and finally water flooding was carried out. After the water content reached 98%, the experiment was stopped and the pressure data was recorded. The second group is water flooding. When the water content reaches 98%, the second stage is displaced by the nanofluid–surfactant binary flooding system (iNanoW1.0−Has5). Finally, the post-water flooding is carried out. When the water content reaches 98%, the experiment is stopped and the pressure data is recorded. The two groups of experiments compare the displacement effect of single surfactant and the ability of the nanofluid–surfactant binary flooding system (iNanoW1.0−Has5) to improve oil recovery. The above displacement experiments were carried out at a constant displacement rate of 0.1 mL/min.

**Figure 8:**
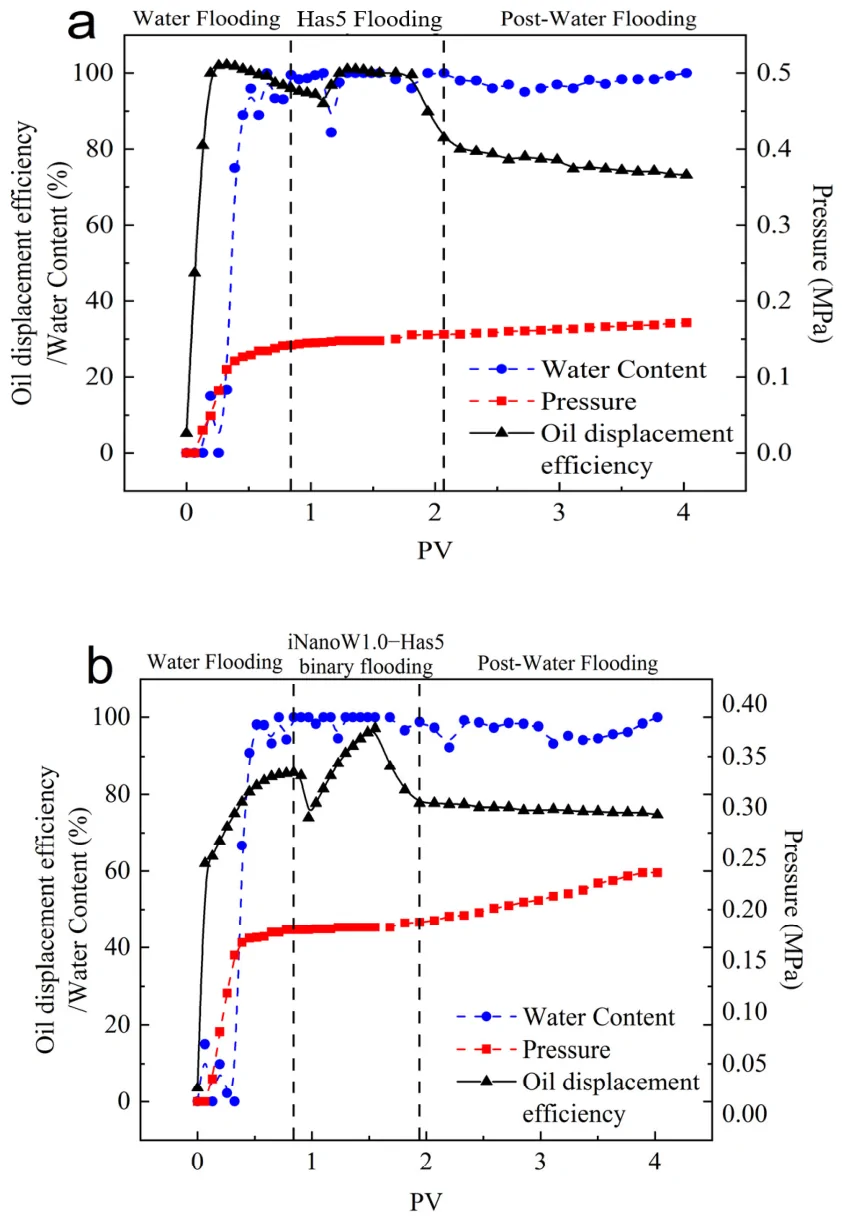
(**a**) Core 3 displacement experiment, (**b**) Core 4 displacement experiment.

As shown in the diagram, with the increase of PV number, the pressure in the water flooding stage gradually increases and then gradually tends to be stable. The reason is that the core permeability is low and the porosity is small, which increases the seepage resistance in the displacement process. When crude oil begins to appear in the produced fluid, it shows that a channel forms inside the core, so that the current pressure can break through the seepage resistance. At this time, the injection pressure begins to change slowly and remains stable overall. The displacement pressure of Core 3 remained basically stable after the injection of a heavy alkylbenzene sulfonate surfactant without adding iNanoW1.0 (Figure 8a). When the surfactant solution opened the seepage channel, the displacement pressure gradually decreased. After the injection of the nanofluid–surfactant binary flooding system (iNanoW1.0−Has5), the displacement pressure of Core 4 increases rapidly (Figure 8b). This is because the presence of iNanoW1.0 blocks some micro pore throats, resulting in a rapid increase in displacement pressure. After the Datong displacement channel, the displacement pressure decreases rapidly. Comparing the displacement pressure between the two shows the highest single-phase surfactant.

The injection pressure of the single surfactant solution is 0.505 MPa. However, the maximum injection pressure of the nanofluid–surfactant binary flooding system (iNanoW1.0−Has5) is only 0.377 MPa, which indicates that the composite system has better injection performance than the single-phase surfactant solution. Due to the relatively smaller particle size of the composite system, its injection performance is greatly improved and the injection pressure will be lower.

The experimental results show that the surfactant or surfactant–nano agent compound system is used after water flooding and its ability to improve oil recovery is limited, i.e. 1.34% and 1.55%, respectively. However, in the post-water flooding stage, the use of the nanofluid-surfactant binary flooding system (iNanoW1.0−Has5) followed by water flooding increased the oil recovery by 13.12%, but the use of surfactant followed by water flooding increased the oil recovery by only 5.11%. This shows that the compound system has better emulsifying ability than the single surfactant. After the compound system enters the pore throat, the crude oil is emulsified, and then the crude oil is carried out by water flooding.

## 4. Conclusions

In the absence of nano-agents, the interfacial tension of Has5 solution (0.2 wt%) is 3 × 10^−3^ mN/m. With the addition of iNanoW1.0, the interfacial tension gradually increases, and the degree of increase is related to the concentration of iNanoW1.0. When the concentration of iNanoW1.0 reaches 0.1%, the interfacial tension can also maintain ultra-low interfacial tension. The particle size of the composite system also decreases with the addition of iNanoW1.0. When the concentration of Has5 is 5000 ppm, the adsorption capacity reaches the maximum value of 1.75 mg/g. With the addition of iNanoW1.0, the adsorption capacity of the chemical agent can be reduced by more than 30% due to the interaction adsorption and competitive adsorption. The adsorption capacity of the system increased with the increase of time, and the increase of adsorption was significantly reduced within the same time difference. Compared with the traditional surfactant flooding or nano-flooding, the nanofluid–surfactant binary flooding system (iNanoW1.0−Has5) system can effectively improve the microscopic swept volume. At the same time, the core displacement test shows that the compound system has better injectivity and lower injection pressure than the surfactant solution. Because the compound system has a good effect of emulsifying crude oil, it can effectively improve the oil recovery rate by 13.12% in post-water flooding. The nanofluid–surfactant binary flooding system possesses advantages over a single surfactant or nano-agent flooding system. It has a smaller particle size to enter the small pore to expand the spread and has a good oil washing effect. The synergistic effect makes the nanofluid–surfactant binary flooding system a perfect candidate for application in low-permeability reservoir recovery.

## Figures and Tables

**Table 1:** Basic parameters of enlarged swept volume core.

Core Number	Length/cm	Diameter/cm	Permeability/mD	Porosity/%
1	5.09	2.48	11.9	13.4
2	5.01	2.52	12.3	12.7

**Table 2:** Basic parameters of conventional displacement core.

Core Number	Length/cm	Diameter/cm	Permeability/mD	Porosity/%
3	5.01	2.51	12.4	13.8
4	5.03	2.50	13.1	13.2

## Data Availability

The original contributions presented in this study are included in the article. Further inquiries can be directed to the corresponding authors.
